# Soybean C2H2-Type Zinc Finger Protein GmZFP3 with Conserved QALGGH Motif Negatively Regulates Drought Responses in Transgenic *Arabidopsis*

**DOI:** 10.3389/fpls.2016.00325

**Published:** 2016-03-18

**Authors:** Dayong Zhang, Jinfeng Tong, Zhaolong Xu, Peipei Wei, Ling Xu, Qun Wan, Yihong Huang, Xiaolan He, Jiayin Yang, Hongbo Shao, Hongxiang Ma

**Affiliations:** ^1^Jiangsu Key Laboratory for Bioresources of Saline Soils, Provincial Key Laboratory of Agrobiology, Institute of Biotechnology, Jiangsu Academy of Agricultural SciencesNanjing, China; ^2^Institute of Botany, Chinese Academy of SciencesNanjing, China; ^3^Huaiyin Institute of Agricultural Sciences of Xuhuai Region in JiangsuHuai'an, China; ^4^Key Laboratory of Coastal Biology and Bioresources Utilization, Yantai Institute of Coastal Zone Research, Chinese Academy of SciencesYantai, China

**Keywords:** Soybean, *GmZFP3*, transgenic *Arabidopsis*, drought response, negatively

## Abstract

Plant response to environmental stresses is regulated by a complicated network of regulatory and functional genes. In this study, we isolated the putative stress-associated gene *GmZFP3* (a C2H2-type Zinc finger protein gene) based on the previous finding that it was one of two genes located in the QTL region between the Satt590 and Satt567 markers related to soybean tolerance to drought. Temporal and spatial expression analysis using quantitative real-time PCR indicated that *GmZFP3* was primarily expressed in roots, stems and leaf organs and was expressed at low levels in flowers and soybean pods. Moreover, *GmZFP3* expression increased in response to polyethylene glycol (PEG) and Abscisic acid (ABA) treatments. In addition, subcellular localization analysis indicated that GmZFP3 was ubiquitously distributed in plant cells. Transgenic experiments indicated that GmZFP3 played a negative role in plant tolerance to drought. Analysis of ABA-related marker gene expression in *Arabidopsis* suggested that GmZFP3 might be involved in the ABA-dependent pathway during the drought stress response. Taken together, these results suggest that soybean GmZFP3 negatively regulates the drought response.

## Introduction

Plants are frequently exposed to a variety of stresses due to environmental changes, and they have evolved efficient mechanisms to adapt to these conditions. Drought is a serious environmental stress factor worldwide (Shao et al., 2009; Harrison et al., 2014). Consequently, it is important to maximize crop yield potential and maintain yield stability under drought conditions to guarantee a food supply for the increasing global population.

The zinc-finger protein (ZFP) family is one of the largest family of transcription factors n plants (Takatsuji, 1999; Iuchi et al., 2001). Among them, the Cys2/His2 (C2H2) zinc finger protein, which typically contains one to four conserved QALGGH motifs in the zinc-finger helices, is a well-characterized eukaryotic transcription factor (Laity et al., 2001) that play various roles in the plant stress response (Kim et al., 2001; Sakamoto et al., 2004; de Lorenzo et al., 2007; Xu et al., 2007; Ciftci-Yilmaz and Mittler, 2008). In *Arabidopsis*, several C2H2-type ZFPs, such as AZF1, AZF2, AZF3, ZAT6, ZAT7, and ZAT10, have been reported to function in the drought and salt stress response (Sakamoto et al., 2000; Ciftci-Yilmaz et al., 2007). Expression of AZFs (AZF1-3) and STZ are strongly induced by dehydration, high-salt, cold stresses, and abscisic acid (ABA) treatment, and they function as transcriptional repressors to increase stress tolerance following growth retardation (Sakamoto et al., 2004). ZAT12 plays a role in ROS and abiotic stress signaling transduction (Davletova et al., 2005). ZAT10 has dual roles, as both overexpression and RNAi largely increased plant tolerance to environmental stresses (Mittler et al., 2006). AZF2 has been identified as an ABA-induced transcriptional repressor during seed germination (Kodaira et al., 2011). However, MPK6 phosphorylation regulates ZAT6 during seed germination under salt and osmotic stress (Liu et al., 2013).

Huang et al. (2009) cloned and characterized rice *DST* (Drought and salt tolerance) and found that it negatively regulated stomatal closure by binding to the TGCTANNATTG element and directly modulated H_2_O_2_ homeostasis-related gene. However, Jan et al. (2013) found that rice CCCH-Type OsTZF1 bound to the RNA poly(u) rich region in an *in vitro* RNA gel electrophoresis assay. Several recent reports used transgenic approaches to show that rice ZFP proteins, such as ZFP179 (Sun et al., 2010), ZFP182 (Zhang et al., 2012), and ZFP36 (Zhang et al., 2014), are involved in the ABA-dependent pathway to regulate the response to drought and salt and oxidative stresses. These reports revealed that zinc finger proteins play an important role in withstanding many stresses and plant growth and development.

Luo et al. (2012) cloned soybean *GsZFP1* from *Glycine soja* and found that it played a crucial role in the plant response to cold and drought stress. They further found that *GsZFP1* over-expression in *Arabidopsis* reduced ABA sensitivity and decreased stomata size under ABA treatment. Kim et al. (2001) found that the C2H2-type protein *SCOF-1* was specifically induced by low temperature and ABA, but not by dehydration or salinity. Furthermore, SCOF-1 interacted with SGBF-1 in a yeast two-hybrid system, suggesting that SCOF-1 functions as a positive regulator of ABRE-mediated *COR* gene expression through protein-protein interactions, which in turn, enhances plant cold tolerance. Yu et al. (2014) reported that soybean GmZF1 enhanced *Arabidopsis* tolerance to cold stress by regulating cold-regulation gene expression.

Specht et al. (2001) identified a WUE (Water Use Efficiency)-related QTL region between the Satt590 and Satt567 markers on chromosome 7 in soybean, and the Glyma07g01900 and Glyma07g05820 gene loci were borders of this QTL region. All 393 genes between these two loci were downloaded using the Perl program and isolated. One such gene was *GmZFP3*, a putative stress-associated gene, was selected for further study. *GmZFP3* was primarily expressed in the root and stem, while GmZFP3 protein was ubiquitously distributed among plant cells. Transgenic experiments indicated that GmZFP3 played a negative role in plant tolerance to drought and that it might be involved in the ABA-dependent pathway during response to drought stress.

## Materials and methods

### Plant materials

The *Glycine max* var. Willimas 82 variety was used to grow seedlings and extract total RNA for *GmZFP3* gene cloning, tissue expression and induced expression analysis experiments. *Arabidopsis* ecotype Col-0 was used for transformation and protoplast preparation and grown in a 7:2:1(v/v/v) mixture of vermiculite: soilrite: perlite under a 16 h light/ 8 h dark regime. The day/light temperature was 23/20°C. Plants were watered every week.

### DNA and RNA isolation

Genomic DNA was extracted from fresh leaves using the cetyltrimethylammonium bromide (CTAB) method. Total RNA was extracted from soybean and *Arabidopsis* samples according to the TRIZOL Kit (Invitrogen,China) manual.

### Gene cloning and sequence analysis

The QTL region sequences between markers Satt590 and Satt567 related to soybean drought tolerance were downloaded from http://www.phytozome.net/cgi-bin/gbrowse/soybean/ using the perl program. The functional annotation of genes was confirmed with BLAST2GO software. The *GmZFP3* gene primers (see Table 1) were designed according to the full-length coding sequence and used to clone the genes from soybean root tissue cDNA using RT-PCR (Reverse transcriptase-polymerase chain reaction).

**Table 1:** **Primer sequences used in this study**.

**Gene**	**Forward primer 5′-3′**	**Reverse primer 5′-3′**	**Purpose**
*GmZFP3*	ATCAACACTCAAACAAAGACGAA	GAGTATGTGTGTCCAAAATCTGC	Semi-RT-PCR and QRT-PCR
*GmZFP3*	CGCGTCGACATGCCtTCTGAAAATT	CGCCCATGGGAGCTTAAGGGACAAG	Subcellular localization in Arabidopsis protoplast
*GmZFP3*	CACCATGCCTTCTGAAAATTTGAA	AGCTTAAGGGACAAGTCAAGCTT	Subcellular localization in tobacco leaf
*GmZFP3*	GGGGACAAGTTTGTC AAAAAAGCAGGCTTCACCATGCCtTCTGAAAATTTGA	GGGGACCACTTTGTACAA GAAAGCTGGGTTGAGCTTAAGGGACAAGTCAAG	Overexpression (OE)
*GmActin*	CGGTGGTTCTATCTTGGCATC	GTCTTTCGCTTCAATAACCCTA	qRT-PCR (internal control)

The Neighbor Joining (NJ) tree of ZFPs from soybean and other plants was performed using MEGA4 software (Tamura et al., 2007).

### RT-PCR and qPCR analysis

The soybean root, stem, and leaf from three different stages, including young seedling stage, flowering stage, and podding stage, were harvested and frozen in liquid nitrogen for RNA extraction. The soybean roots were collected from plants treated with PEG6000 for 0, 2, 4, and 12 h or with 100 μM ABA for 10, 20, 30, 45, 60, 90, and 120 min. The method was the same for all samples, including *Arabidopsis*. Total RNA from all samples was isolated using TRIzol® reagent (Invitrogen) following the manufacturer's instructions. Single-stranded cDNA was synthesized using 2 μg total RNA and Oligod(T)18 primer with the Takara RT-PCR system in a total volume of 25 μl according to the manufacturer's protocol (TaKaRa Bio Inc.) and used for RT-PCR and qRT-PCR analysis. RT-PCR and qPCR analysis was performed as described previously (Zhang et al., 2013). One microliter of the cDNA mix was used as a template in a 25 μL PCR reaction volume. The RT-PCR condition was 94°C, 3 min, and 27 cycles of 94°C, 30 s; 55°C, 30 s; 72°C, 0.5 min, with a final extension of 10 min at 72°C. PCR products were separated on 1% agarose gel containing ethidium bromide and were photographed. For qPCR analysis, equal amount of cDNA prepared was analyzed by quantitative Real-Time PCR using a Roche 2.0 Real-Time PCR Detection System with the SYBR Green Supermix (Takara).

The assays were repeated three times. Arabidopsis primers are listed in Table 2.

**Table 2:** **List of primers for stress-related genes and internal control in *Arabidopsis***.

**Gene**	**Locus**	**Forward primer (5′-3′)**	**Reverse primer (5′-3′)**
*CCA1*	AT2G46830	TGTGGCTCAAACACTCCG	GCAATTCGACCCTCGTCA
*LHY*	AT1G01060	AAGTCTCCGAAGAGGGTC	CATGTTCCAACACCGATC
*UGT71B6*	AT3G21780	TTTGATGGAGCAAGACAG	GTTTCCGACCAAGCAATA
*DREB2A*	AT5G05410	AACAGAAGGAGCAAGGGAT	ACATCGTCGCCATTTAGG
*MYB2*	AT2G47190	ACGCCCAATCATTACCCA	AACCTGACCCGTTCACCA
*PAD3*	AT3G26830	TATGCGATGGGTCGTGAT	TTTGGCTTCCTCCTGCTT
*RCI3*	AT1G05260	AGCCTCAACGATAACAAG	TGAAACAGACCTCTACGC
*LTP3*	AT5G59320	ATGTGGCACAGTGGCAGGTA	CTTGTTGGCGGTCTGGTG
*At5g02840*	NM_120362	TATTCCACCAGAAGATGA	CACTCCCAATGAAGTTAT
*AtActin2*	NM_112764.3	CCTCCGTCTTGACCTTGC	AGCGATACCTGAGAACATAGTG

### Subcellular localization

PCR-generated *Sal1-Nco1* and *Hind III- BamH1* fragments containing the open reading frame of *GmZFP3*, respectively, were subcloned in-frame upstream of the GFP gene in the pJIT166GFP plasmid. All constructs were validated by sequencing. The primer sequences are listed in Table 1.

*Arabidopsis* leaf protoplasts were isolated according to Yoo et al. (2007). The two resulting fusion constructs or empty control vector (p35S::GFP) were introduced into *Arabidopsis* protoplasts by the PEG4000-mediated method (Abel and Theologis, 1994). After incubation of transformed *Arabidopsis* protoplasts for 18−24 h at room temperature, GFP signal was detected by confocal fluorescence microscopy (Zeiss, LSM510 Meta, Carl Zeiss AG).

To further validate GmZFP3 localization, full-length *GmZFP3* CDS lacking a stop codon (primers used are listed in Table 1) were amplified and cloned into the pMDC83 destination vector using the Gateway™ method. The in-frame GFP fusion constructs were transformed into the *Agrobacterium tumefaciens* GV3101 strain by electroporation and injected into the tobacco leaf. GFP signal was examined by confocal fluorescence microscopy (Zeiss, LSM510 Meta, Carl Zeiss AG). Nuclei were stained with DAPI (4′,6-diamidino-2-phenylindole).

### Transformation of *Arabidopsis*

The *Agrobacterium* strain containing the pearlygate103-GmZFP3 construct was grown on Luria-Bertani plates containing 50 mg/L kanamycin and 100 mg/L rifampicin at 28°C for 48 h. A single colony was transferred to 5 mL DYT medium containing the same antibiotics and was cultured overnight at 28°C with vigorous shaking (180 rpm). The overnight culture was added to 500 mL of the same medium and cultured overnight to an OD_600_ of 0.5 to 1. *Agrobacterium* cells were harvested by centrifugation for 15 min (4000 rpm, 4°C) and re-suspended in infiltration medium (5.0% Suc and 0.05% Silwet L-77). *Arabidopsis* plants were transformed by the floral dip method (Clough and Bent, 1998). Transgenic plants were selected on selective medium containing kanamycin. Transgenic plants were transferred to soil and grown until seed harvest.

### Architecture parameter measurements

Before treatment, the total root length, water loss rate from 0 to 24 h, stomatal width/length and density were measured in *GmZFP3*-overexpressing transgenic *Arabidopsis* plants and WT control plants. After air drought treatment application, including no watering and rewatering, the phenotype change of transgenic, and wild type were recorded using camera (Canon,Japan). The experiments were repeated independently at least three times.

### SEM observation and statistical analysis

Leaves from ~3-week-old non-transgenic and transgenic GmZFP3 *Arabidopsis* plants were stripped and serially dehydrated for 30 min each in 30, 50, 70, 80, 90, and 100% ethanol solutions. They were then placed in isoamyl acetate three times for 30 min each. Thereafter, samples were freeze-dried (Hitachi ES-2030, Tokyo, Japan) and sputter-coated with silver using an ion sputter (Hitachi E-1010/E-1020), then the specimens were observed by SEM (Hitachi S-3000N). Each specimen was observed at an accelerating voltage of 15 KV, and images were stored as TIF files. The width/length, density, and open/close status of stoma were measured using WinRhizo software (Regent Instruments, Montreal, QC, Canada).

### Statistical analysis

Data were analyzed using an one-way ANOVA test in Microsoft Excel. Significant differences among means were determined by the LSD (Least Significant Difference),^*^ at *P* < 0.05 and ^**^ at *P* < 0.01.

## Results

### Isolation and characterization of GmZFP3

**Figure 1:**
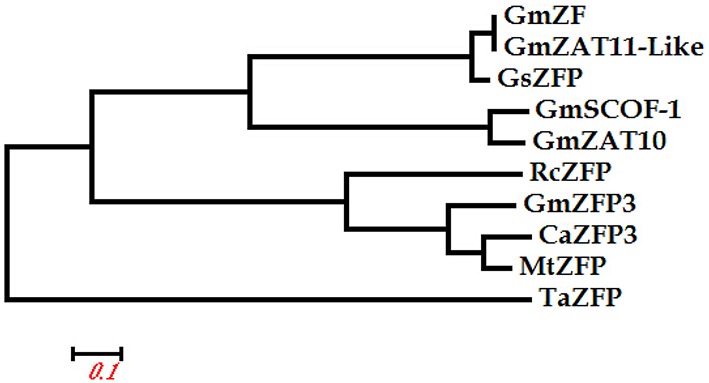
**Phylogenetic tree of GmZFP3 and other ZFP proteins from *Triticum aestivum* (TaZFP accession number: AEE81066), *Glycine max* (GmZF: DQ055134; GmSCOF-1: U68763; GmZAT11-Like: XM_003547379; GmZAT10: NM_001267693), *Glycine soja* (GsZFP:FJ417330), *Ricinus communis* (RcZFP:XM_002515403), *Medicago truncatula* (MtZFP:XM_003604157), and *Cicer arietinum* (CaZFP3:XM_004515472)**. The GmZFP3 cloned in this paper had the highest similarity with ZFP3 proteins from other plants. Thus, ZFP was designated GmZFP3.

**Figure 2:**
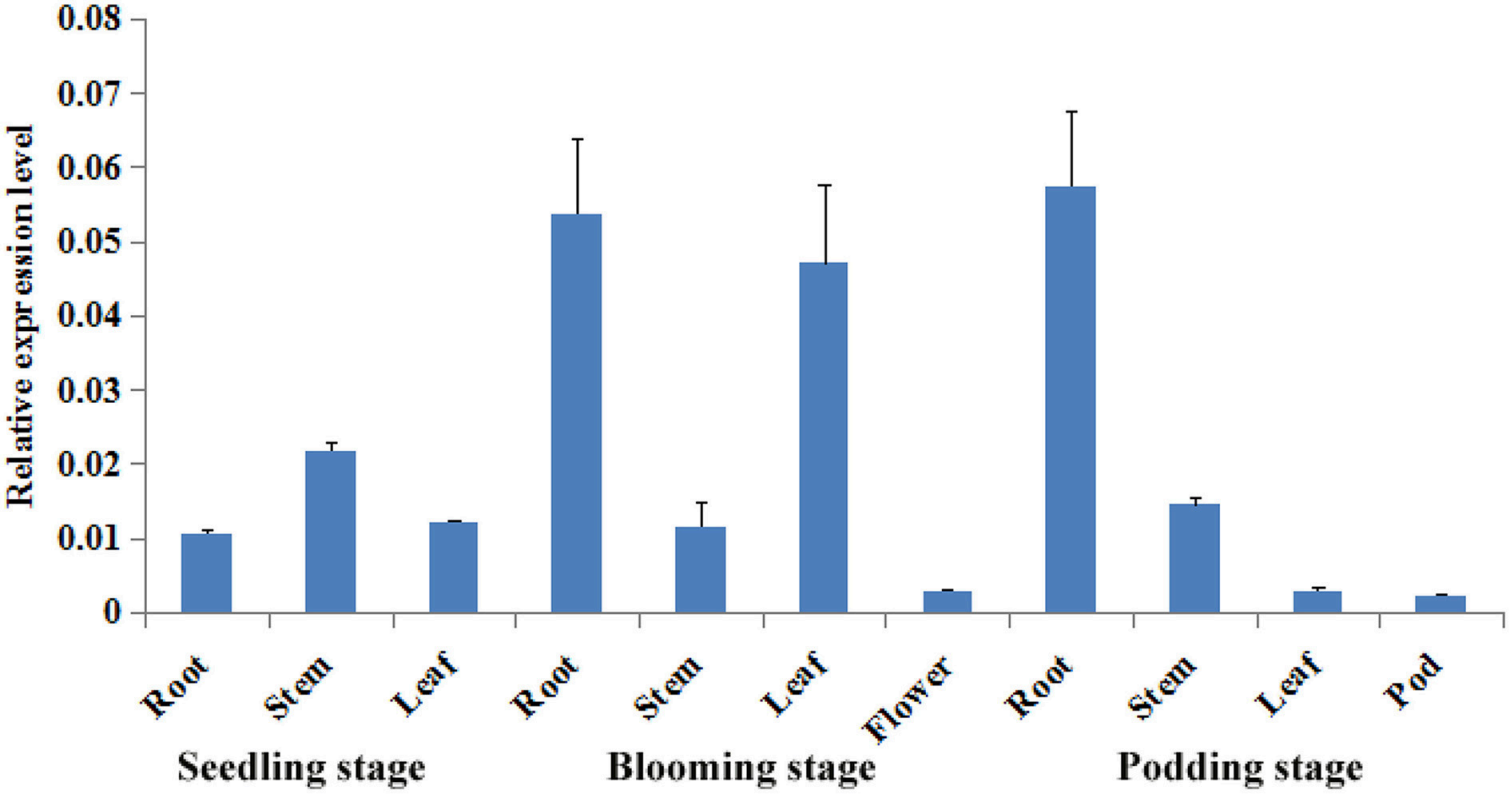
**The temporal and spatial expression pattern of *GmZFP3* in different organs and stages**. The expression pattern of *GmZFP3* in root, stem, leaf, flower and pod at seedling, flowering, and podding stages, respectively using quantitative RT-PCR. The picture showed that *GmZFP3* expressed all the tested tissues including, root, stem, and leaf at young seedlings, and increased abundance in stem and leaf at blooming stage, but weak in flower, and kept the similar expression level in root and stem, but decreased in leaf and weak in pod at podding stage. The relative expression level at Y axis indicates the expression of *GmZFP3*/*GmActin*.

We cloned and sequenced the putative drought-associated gene *GmZFP3* from soybean. Sequence analysis indicated that the GmZFP3 protein contains one zinc finger motif and one conserved QALGGH motif. Phylogenetic tree analysis of GmZFP3 and other ZFP proteins from other plants, including *Triticum aestivum, G. soja, Ricinus communis, Medicago truncatula*, and *Cicer arietinum*, showed that GmZFP3 was most similar to ZFP3 proteins from *M. truncatula* and *C. arietinum* (Figure 1). Thus, we designated GmZFP as GmZFP3. We then analyzed *GmZFP3* expression in various tissues/organs at different developmental stages of soybean cv. Willimas 82 by qRT-PCR. *GmZFP3* transcript was expressed in the root, stem, and leaf of young seedlings. Its expression increased in the stem and leaf but was weak in the flower at the blooming stage. Finally, it was primarily expressed in the root and stem at the podding stage (Figure 2).

**Figure 3:**
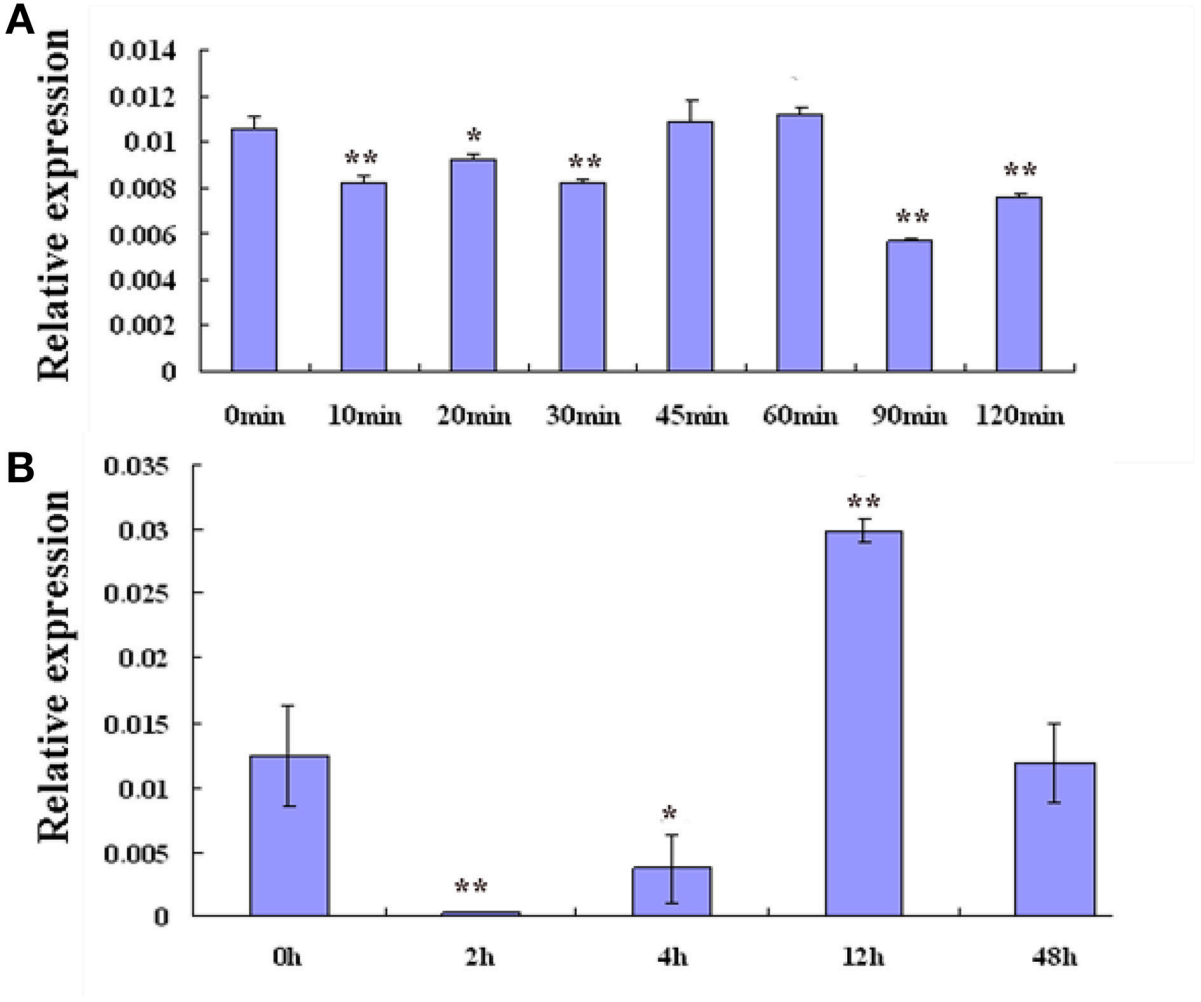
**The expression pattern of *GmZFP3* in soybean roots under PEG6000 (20%) and 100 μM ABA treatments. (A)** The expression pattern of *GmZFP3* in soybean roots under 100 μM ABA for 10, 20, 30, 45, 60, 90, or 120 min. **(B)** The expression pattern of *GmZFP3* in soybean roots under PEG6000 (20%) for 2, 4, 12, or 48 h. ^*^p < 0.05 level; ^**^p < 0.01.The relative expression level at Y axis indicates the expression of *GmZFP3*/*GmActin*.

To test whether drought stress induced *GmZFP3* expression, we treated the roots of soybean seedlings with PEG and ABA, then analyzed *GmZFP3* expression by quantitative real-time RT-PCR. ABA treatment (100 μM) initially significantly suppressed *GmZFP3* expression after 30 min treatment (*p* < 0.05), but then it increased back to its initial expression level from 45 to 60 min, then continuously decreased from 90 to 120 min (Figure 3A). However, *GmZFP3* expression decreased within 2 h of PEG-6000 (20%) treatment, then its mRNA expression continuously increased from 4 to 12 h and reached a maximum at 12 h. Finally, its expression decreased from 12 to 48 h (Figure 3B). Taken together, these data suggest that *GmZFP3* expression changed in response to PEG and ABA.

### GmZFP3 subcellular localization

**Figure 4:**
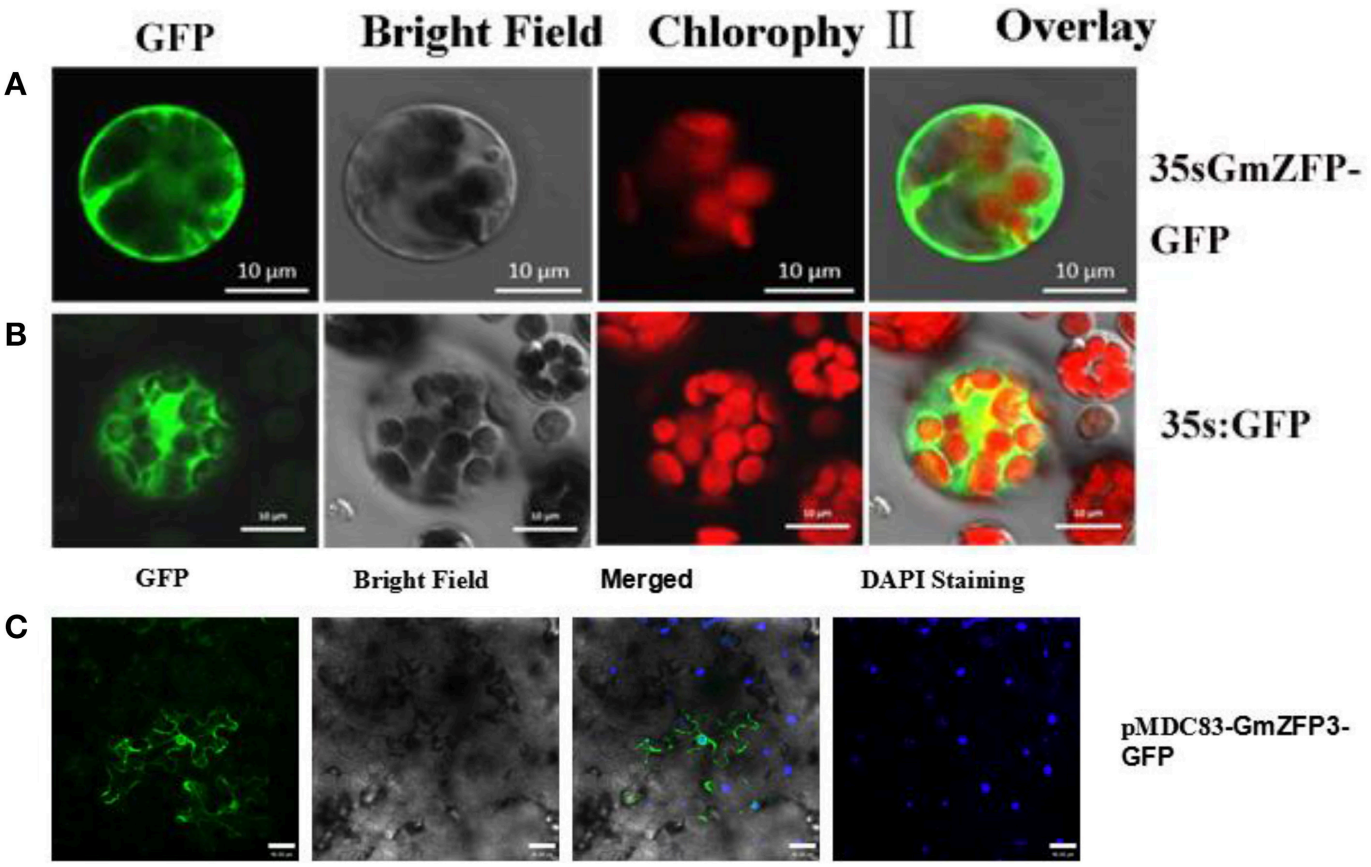
**Subcellular localization of GmZFP3 protein in *Arabidopsis* protoplasts and in tobacco leaf epidermal cells**. The GmZFP3-green fluorescent protein (p35S::GmZFP3-GFP) fusion construct was generated by inserting GmZFP3 into pJIT166-GFP and pMDC83 vector without a termination codon to create an in-frame fusion between the CDS and GFP. p35S::GmZFP3-GFP and the GFP control plasmid (p35S::GFP) were transformed into *Arabidopsis* protoplasts by the PEG4000-mediated method respectively. In addition, the pMDC-83-*GmZFP3* construct was injected into the tobacco leaf epidermical peel cell respectively. **(A)** GmZFP3 distributed throughout the cell, especially nucleus **(B)**. The GFP control distributed throughout the whole cell (Con). Scale bars = 10 μm. **(C)** Subcellular localization of GmZFP3 in tobacco leaf epidermal cells. GmZFP3 distributed throughout the cell, especially the plasma membrane and nucleus. Nuclei were stained with DAPI. Scale bars = 40 μm.

To examine GmZFP3 protein localization, we fused its coding sequences N-terminal to green fluorescent protein (GFP). We then transformed the fused proteins into *Arabidopsis* leaf protoplasts. The GmZFP3::GFP fluorescence signal was distributed throughout the cell, including the nucleus (Figure 4A), similar to the ubiquitous distribution of free GFP (Figure 4B). When we used the injected tobacco leaf method, GmZFP3 also distributed throughout the cell, especially the plasma membrane and nucleus (Figure 4C).

### GmZFP3 drought response

**Figure 5:**
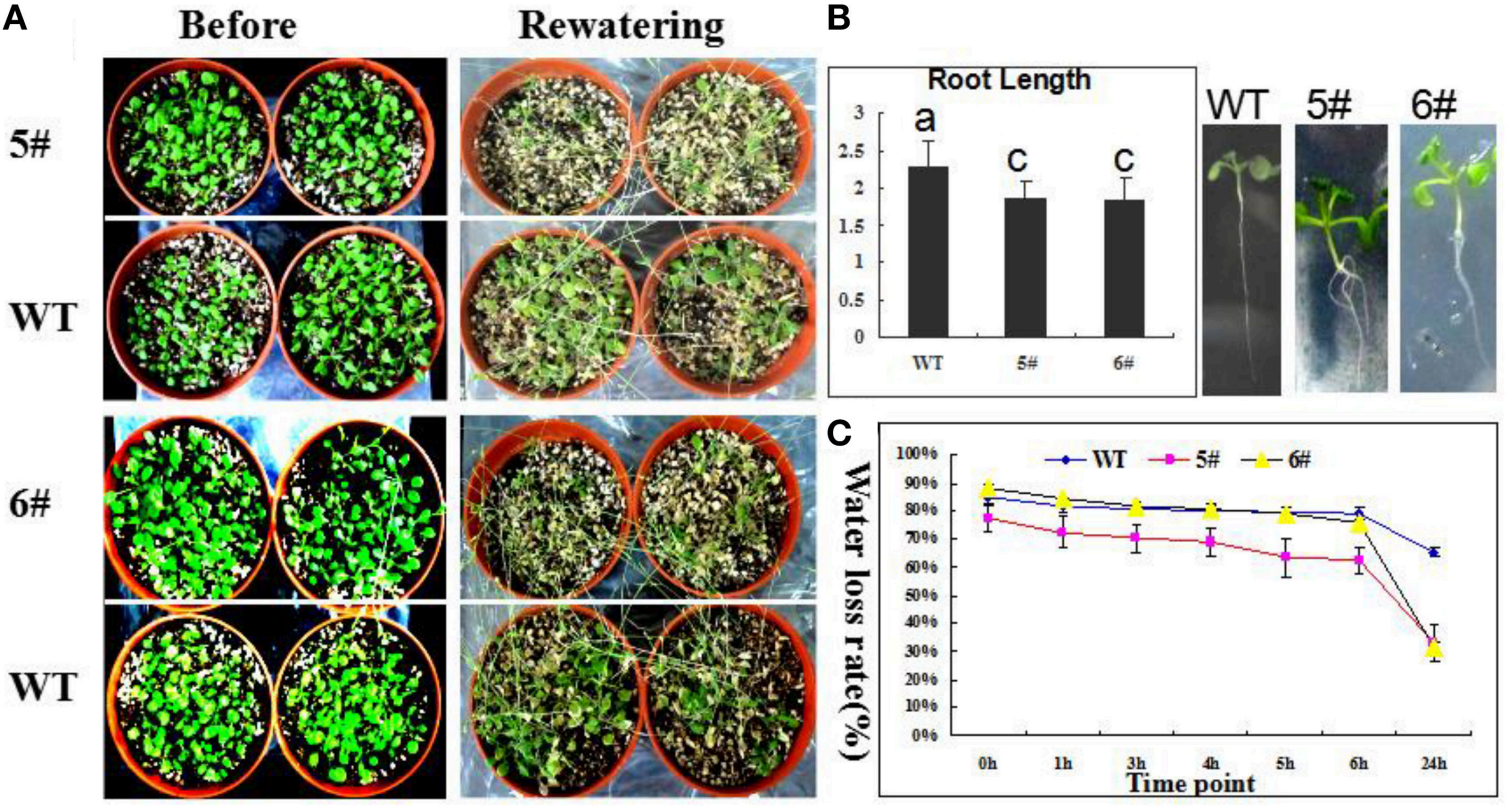
**Performance of transgenic *GmZFP3*- overexpressing *Arabidopsis* plants under drought stress**. **(A)** The response of transgenic plants and wild type (WT) to drought stress. The transgenic plants (#5 and #6) showed the more sensitive to drought treatment compared with the WT plants. **(B)** The root length of transgenic was significantly shorter than that of WT. The roots of transgenic plants (#5 and #6) had the shorter primary root compared with the WT plants. **(C)** The water loss rate under air condition in transgenic and WT. When detached leaves from transgenic and WT plant were placed the air condition, the transgenic plants (#5 and #6) showed a faster water loss compared with the WT plants.

To further validate GmZFP3 function in drought stress, we did not water 4-week-old transgenic *Arabidopsis* seedlings for 3 weeks and used wild type (WT) *Arabidopsis* plants as controls. Seedlings from transgenic *GmZFP3 Arabidopsis* lines #5 and #6 grew worse compared to controls (Figure 5A). The survival rate of transgenic lines was significantly lower than that of wild type(WT), suggesting that *GmZFP3* overexpression did not improve drought tolerance, likely due to the decreased root length and increased water loss rate in transgenic plants (Figures 5B,C).

**Figure 6:**
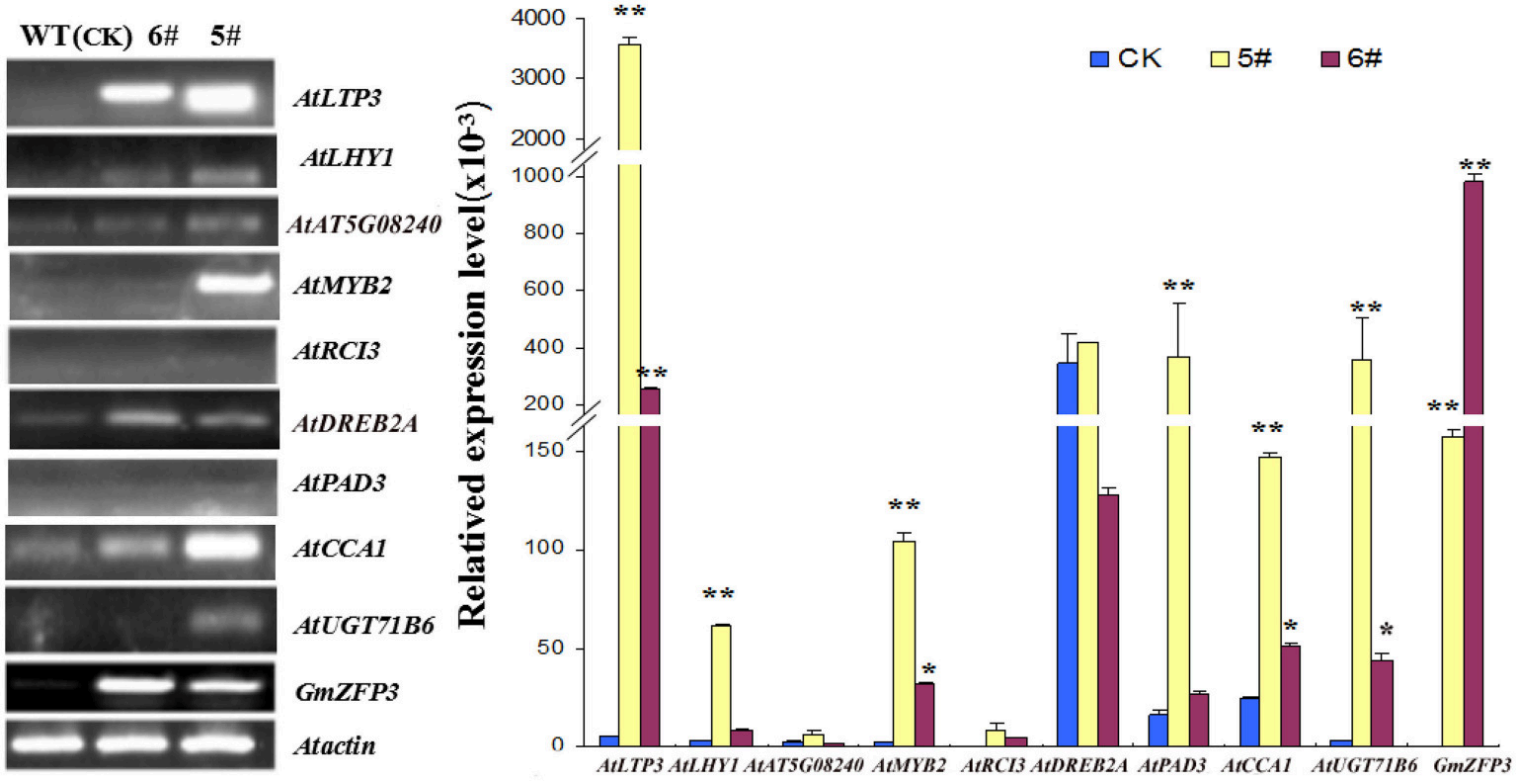
**RT-PCR and qRT-PCR results for the relative transcript abundance of nine stress-related genes in transgenic *GmZFP3*-overexpressing plants**. Leaves of 2-week-old transgenic *GmZFP3*-overexpressing *Arabidopsis* seedlings. The *Actin* (NM_112764.3) gene was used as an internal control. The nine genes, including *AtLTP3, AtLHY1, AtAT5G08240, AtMYB2, AtRCI3, AtDREB2A, AtPAD3, AtCCA1*, and *AtUGT71B6* related to abiotic stress or ABA signaling pathway were selected for comparative analysis of differential expression between transgenic plants(#5 and 6#) and WT, among of which six genes showed the significant different expression except for *AtDREB2A, AtRCI3*, and *AtAT5G08240*. The relative expression level at Y axis indicates the genes expression above mentioned from *Arabidopsis*/*GmActin* respectivey. ^*^indicated significantly < 0.05 level, ^**^ indicated significantly < 0.01 level.

**Figure 7:**
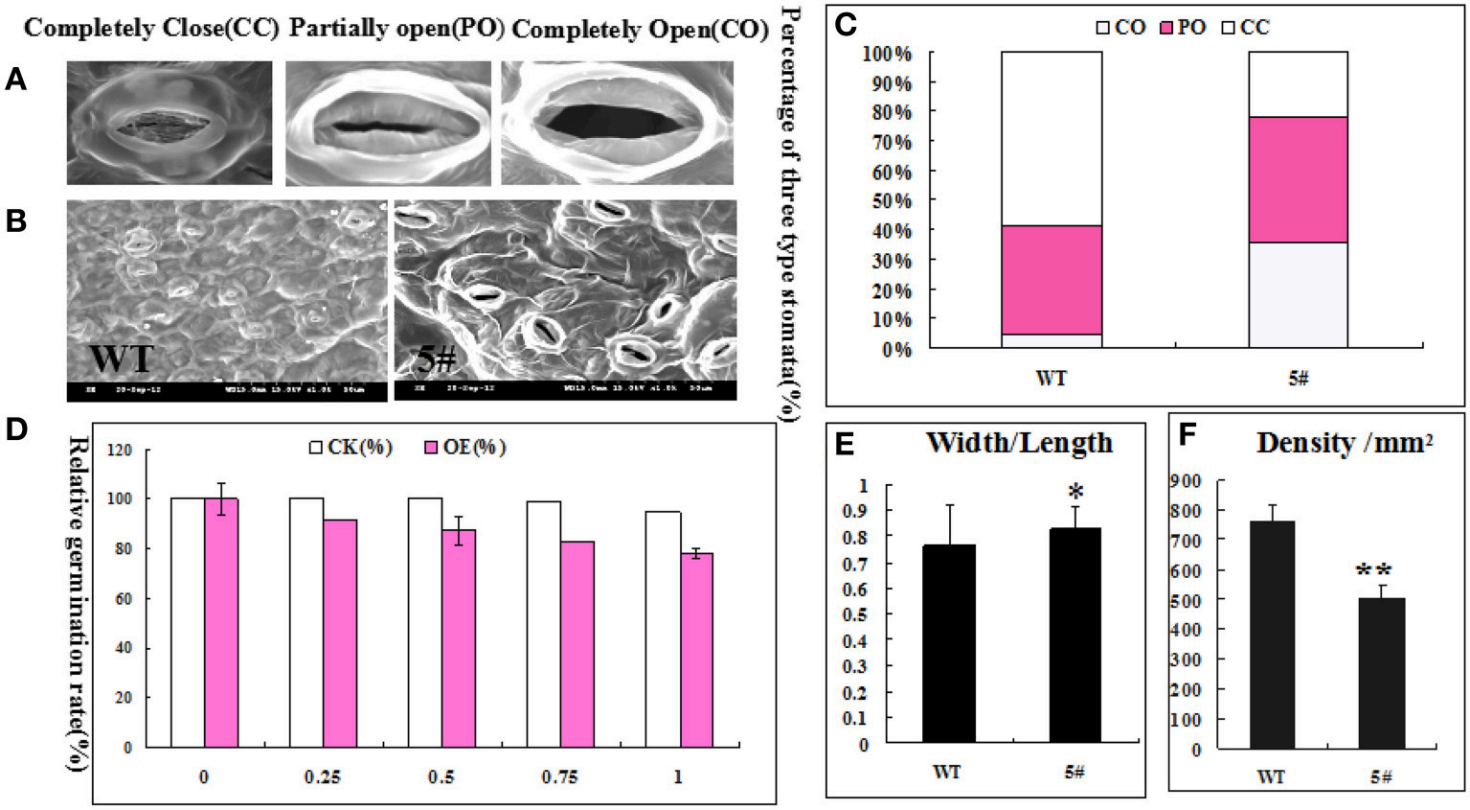
**The relationship between transgenic plant ABA-induced drought sensitivity and stomata**. **(A)** Environmental scanning electron microscopy images of three stomatal levels. **(B)** Scanning electron microscopy images of transgenic line #5 and wild-type. **(C)** The percentage of the three types of stomatal opening in transgenic line #5 and wild-type. **(D)** The germination rate of transgenic line #5 and wild-type (WT) under different ABA concentrations. **(E)** The stomatal width/length in transgenic line #5 and wild-type. **(F)** The stomatal density in transgenic line #5 and wild-type. Significant difference at ^*^P < 0.05 and ^**^P < 0.01 level.

To determine the function of GmZFP3 in drought response, we performed RT-PCR and qRT-PCR for nine genes related to stress/or ABA signaling pathway in transgenic lines #5 and #6, including *DREB2A, LHY1, AT5G02840, MYB2, RCI3, PAD3, CCA1*, and *UGT71B6*. Among the nine genes tested, six genes related to the ABA-dependent signaling pathway were up-regulated, with the exception of *DREB2A* related to the ABA-independent pathway and *AT5G02840* compared to wild-type (Figure 6). Because of the higher expression in transgenic line #5, we measured the relative germination rate, stoma width/length, and density for line #5 under different ABA concentrations. We found that leaf stoma in transgenic plants were primarily completely open (CO), while stoma in wild-type plants were primarily completely closed (CC) (Figures 7A–C). The relative germination rate was significantly lower in GmZFP3-overexpressing plants compared to wild-type after ABA treatment (Figure 7D), suggesting that GmZFP3-overexpressing plants were sensitive to ABA. Moreover, the width/length was significantly higher compared to wild-type, but the stoma density was significantly decreased under the same conditions (Figures 7E,F). Taken together, these data suggest that GmZFP3 functioned in the ABA-dependent pathway during the drought response.

## Discussion

Specht et al. (2001) identified a WUE (Water Use Efficiency)-related QTL region between the Satt590 and Satt567 markers on chromosome 7 in soybean. We searched the http://www.phytozome.net/cgi-bin/gbrowse/soybean/ website using these two markers as probes and identified the Glyma07g01900 and Glyma07g05820 gene loci between these two markers. We downloaded and isolated all 393 genes within these two loci using the Perl program. Function categorization analysis showed that the number of other function was 146(37%), enzyme was 95(24%), unknown function was 90(23%), transcription factor was 37(9%), and zinc finger was 13(3%). GO classification, including biological process, cellular component, and molecular function, indicated that oxidation reduction and regulation of transcription, mitochondrial, and ATP binding were primarily related to this QTL (Figures S1, S2A–C).

Based on the many reports about plant drought tolerance, we selected two aquaporin gene TIP (TIP2;3, Glyma07g02060) and NIP (Glyma07g02800), six transcription factors HD (Homeodomain)-zip (Glyma07g01940), ERF (Glyma07g02380), bZIP38 (Glyma07g02050), Zinc Finger Protein (ZFP3, Glyma07g02880), bHLH (Glyma07g02120), and WRKY27 (Glyma07g02630) as candidate genes for soybean drought tolerance. Among them, the *GmTIP2;3* and *GmZFP3* genes showed similar spatial and temporal expression patterns after 10%PEG6000 or 100 μM ABA treatments for different time periods. More importantly, when we co-transformed pEarlygate103-HD-zip(OE), GmERF(OE), GmbZIP38(OE), GmZFP3(OE), bHLH(OE), and pGUS-*GmTIP2;3*-promoter(Zhang et al., 2016), only GmZFP3 could altered and inhibited GUS expression, leading to the lack of blue color(Data not shown), indicating that GmZFP3 inhibited the expression of *GmTIP2;3*.

In addition, GmZF1 contained a conserved QALGGH motif, which has been previously been shown to enhance *Arabidopsis* tolerance to cold stress (Yu et al., 2014). However, studies on *Arabidopsis* transformed with GsZFP1 lacking the typical QALGGH motif revealed that it played an important role in withstanding cold and drought stresses (Luo et al., 2012), suggesting that the QALGGH motif in the GsZFP1 protein was not necessary for adaptation to abiotic stress in wild soybean. In our study, GmZFP3 also had a plant-specific typical QALGGH motif, suggesting that this motif was crucial for plant response to abiotic stresses in cultivated soybean.

Xie et al. (2009) found that soybean trihelix transcription factors GmGT-2A and GmGT-2B improved plant tolerance to abiotic stresses in transgenic *Arabidopsis*, as the expression of *CCA1, LHY1, MYB2*, and *AT5G02840* was down-regulated in transgenic plants compared to WT, while the expression of *PAD3, LTP3, RCI3, UGT71B6*, and *DREB2A* was up-regulated. In this study, we selected these nine stress-responsive genes for further quantitative real-time PCR analysis in transgenic and WT plants. Six genes, including *LHY1, MYB2, CCA1, PAD3, LTP3*, and *UGT71B6*, were up-regulated in transgenic lines expressing *GmZFP3*.*RCI3*, while *AT5G02840* expression did not significantly change between transgenic and WT plants, in contrast to the report by Xie et al, especially for *LHY1, MYB2*, and *CCA1*, implying a difference in gene function. *LTP3, PAD3*, and *UGT71B6* are involved in ABA responses (Arondel et al., 2000; Priest et al., 2006; Kaliff et al., 2007). RCI3 encodes a peroxidase, and its overexpression conferred dehydration and salt tolerance (Llorente et al., 2002). DREB2A acts as a trans-acting factor in the signal transduction pathway under dehydration conditions, and dehydration induced its expression (Liu et al., 1998). *DREB2A* expression does not activate downstream genes under normal growth conditions. However, overexpression of its constitutively active form leads to drought stress tolerance and slight freezing tolerance (Sakuma et al., 2006). *LHY1, CCA1, At5g02840*, and *MYB2* have been found to be responsive to stress and/or ABA (Boxall et al., 2005). In our study, *GmZFP3*-overexpressing *Arabidopsis* plants were more sensitive to drought stress than wild-type plants, consistent with *AtMYB2* function, whose expression was induced by dehydration, high salt stress or exogenous ABA (Urao et al., 1993). However, transgenic plants overexpressing *MYB2* were more sensitive to ABA and showed stress tolerance, while co-expression of *AtMYB2* and *AtMYC2* conferred moderate stress tolerance (Abe et al., 2003). The seed germination rate of transgenic *GmZFP3* plants was influenced by ABA and decreased as ABA concentrations increased. In addition, we observed the stomata by SEM to gain further insight into the mechanism of GmZFP3 during stress. Stomatal aperture (width/length) of transgenic plant leaves under drought conditions was significantly larger than wild-type, and the percentage of completely open (CO) stoma in transgenic plants was higher than wild-type. However, the stomatal density was lower in transgenic plants compared to wild-type. Huang et al. (2009) examined the percentage of three types of stomata, stomatal density, and stomatal conduction between ZH11 and *dst* mutants and found that the completely open level, stomatal density, and aperture in mutants was lower than in wild-type, which was consistent with the positive role under drought stress in *dst* mutants. Therefore, the *GmZFP*-overexpressing plants might lose large volumes of water by increasing the width/length and number of completely open stoma, but not the density, leading to drought stress sensitivity. Moreover, stomatal development and change are closely related to ABA (Trejo et al., 1993). By examining the expression pattern of marker genes related to stress or ABA and the effect of ABA on germination rate, we conclude that the function of GmZFP3 under drought stress involves the ABA pathway but not the ROS pathway.

## Author contributions

DZ, PW, and LX conceived, designed and conducted the experiments. ZX, XH, QW, JY, and YH analyzed the data and results. DZ, HS, and JT wrote the manuscript. HS and HM monitored the experiments and critically commented on the manuscript. All authors read and approved the final manuscript.

### Conflict of interest statement

The authors declare that the research was conducted in the absence of any commercial or financial relationships that could be construed as a potential conflict of interest.

## Supplementary material

Figure S1 **The results of function categorization of 393 genes between the Glyma07g01900 and Glyma07g05820 loci on soybean chromosome 7**. Most of them belong to other (146), the enzyme is 95, unknown function is 90 and 37 transcription factors respectively.

Figure S2 **The results of BLAST2GO analysis of 393 genes between the Glyma07g01900 and Glyma07g05820 loci on soybean chromosome 7. (A)** Concerning the biological process, the top categories with the highest number were regulation of transcription (23), oxidation reduction (13), and amino acid phosphorglution (9), respectively. **(B)** Concerning the molecular function, the top categories with the highest number were ATP binding (15), transcription factor activity (13), iron binding (11), and binding (10), respectively. **(C)** Concerning cell component, the top categories with the highest number were mitochondrion (28), plastid(19), membrance (18), and nucleus(18), respectively.

## Abbreviations

PEG: Polyethylene glycol
GFP: Green Fluorescent Protein
ZFP: Zinc-finger protein
CTAB: hexadecyltrimethylammonium bromide
QTL: quantitative trait locus
RT-PCR: Reverse Transcription-Polymerase Chain Reaction
QRT-PCR: Quantitative reverse transcriptase Chain Reaction
ABA: Abscisic acid
SEM: Scanning electron microscope.
