# Supplementary figures and images for: Soybean C2H2-Type Zinc Finger Protein GmZFP3 with Conserved QALGGH Motif Negatively Regulates Drought Responses in Transgenic Arabidopsis
Source: Front Plant Sci. 2016 Mar 18;7:325. doi: 10.3389/fpls.2016.00325 (PMC4796006; doi:10.3389/fpls.2016.00325)

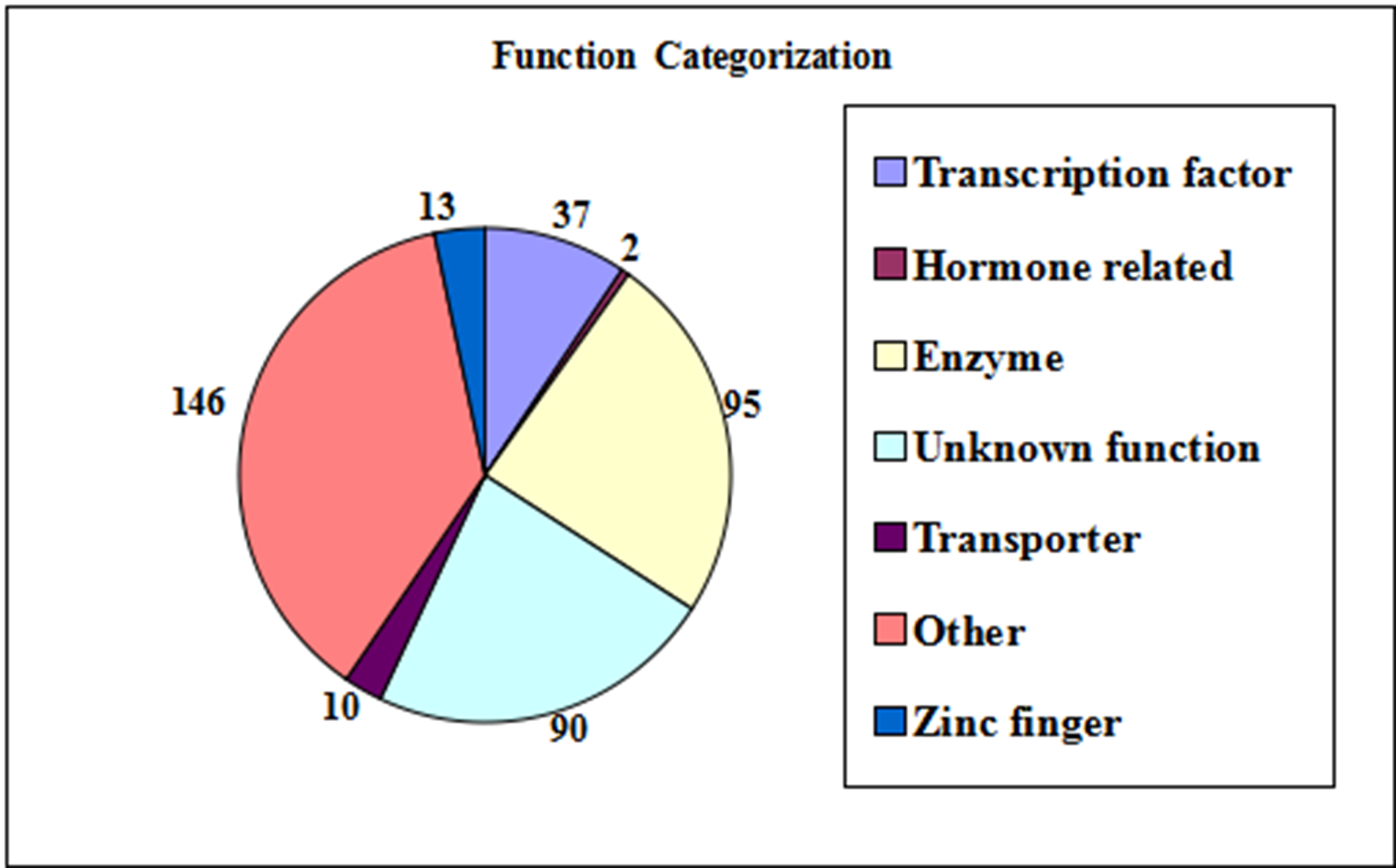

Supplement: Figure S1 — The results of function categorization of 393 genes between the Glyma07g01900 and Glyma07g05820 loci on soybean chromosome 7. Most of them belong to other (146), the enzyme is 95, unknown function is 90 and 37 transcription factors respectively. [file Image1.TIF]

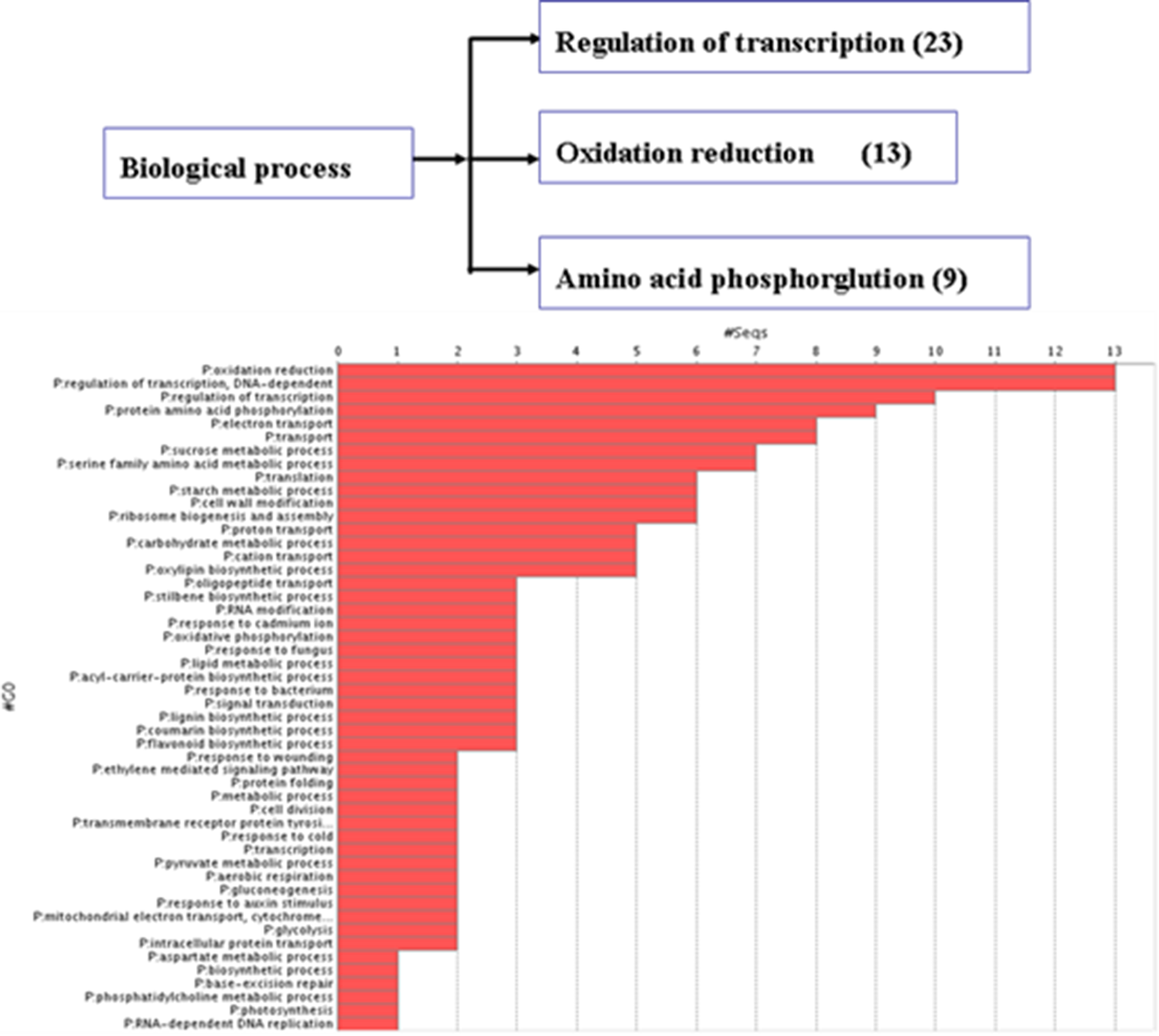

Supplement: Figure S2 — The results of BLAST2GO analysis of 393 genes between the Glyma07g01900 and Glyma07g05820 loci on soybean chromosome 7. (A) Concerning the biological process, the top categories with the highest number were regulation of transcription (23), oxidation reduction (13), and amino acid phosphorglution (9), respectively. (B) Concerning the molecular function, the top categories with the highest number were ATP binding (15), transcription factor activity (13), iron binding (11), and binding (10), respectively. (C) Concerning cell component, the top categories with the highest number were mitochondrion (28), plastid(19), membrance (18), and nucleus(18), respectively. [file Image2.TIF]

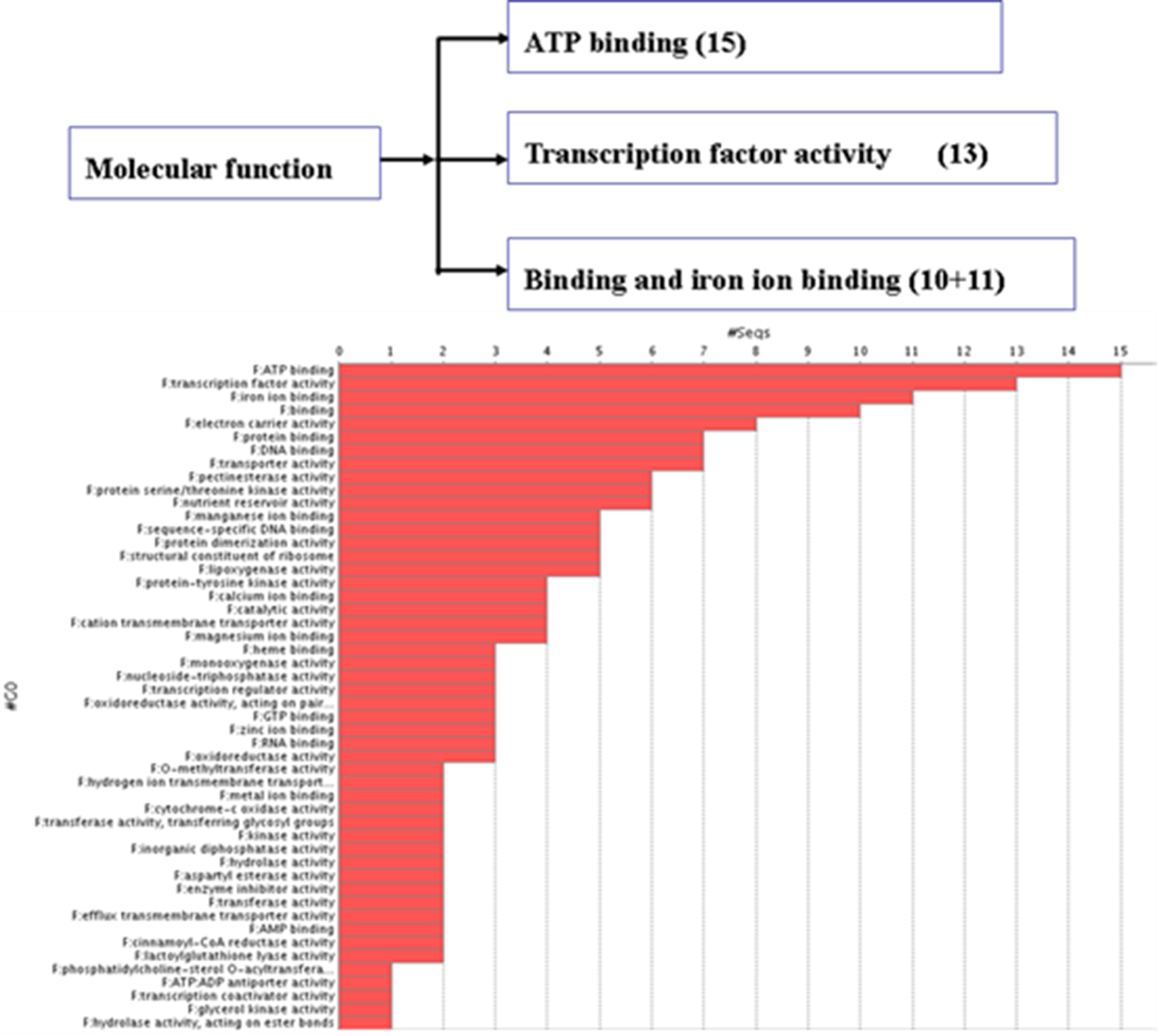

Supplement: Supplementary file 3 [file Image3.TIF]

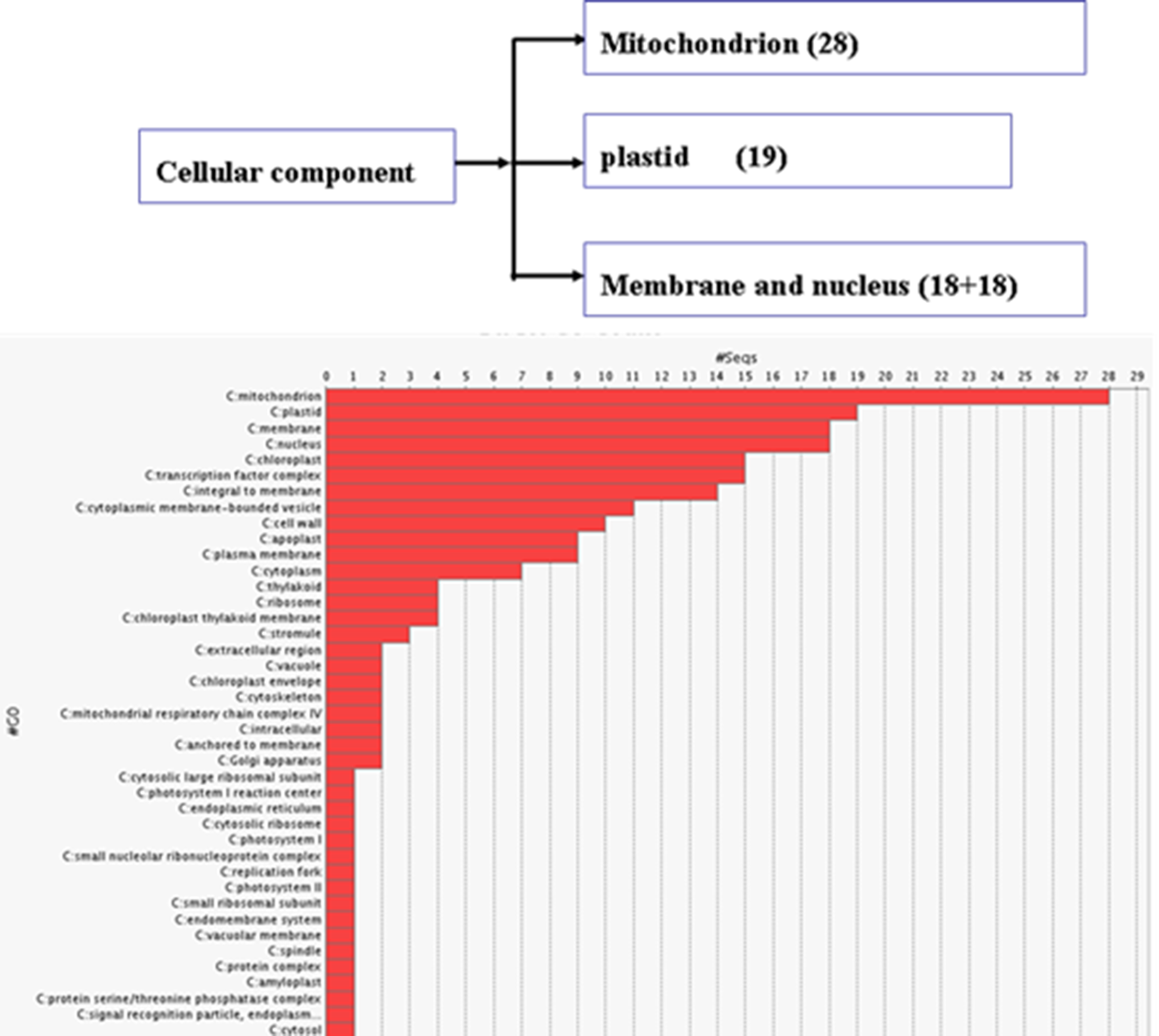

Supplement: Supplementary file 4 [file Image4.TIF]
